# A solid state electrolyte based enzymatic acetone sensor

**DOI:** 10.1038/s41598-024-66498-9

**Published:** 2024-07-04

**Authors:** Yusra M. Obeidat, Nour Bany Hamad, Abdel Monem Rawashdeh

**Affiliations:** 1 Department of Electronics Engineering, Hijjawi Faculty for Engineering Technology, Yarmouk University, Irbid, Jordan; 2 Department of Chemistry, Faculty of Science and Arts, JUST University, Irbid, Jordan; 3 Department of Chemistry, Faculty of Sciences, Yarmouk University, P.O. Box 566, Irbid, Jordan

**Keywords:** Acetone, Amperometry, Cyclic voltammetry, Sensor, Nafion, Enzymatic, Electrochemistry, Biochemistry, Electrochemistry, Electrocatalysis

## Abstract

This paper introduces a novel solid-state electrolyte-based enzymatic sensor designed for the detection of acetone, along with an examination of its performance under various surface modifications aimed at optimizing its sensing capabilities. To measure acetone concentrations in both liquid and vapor states, cyclic voltammetry and amperometry techniques were employed, utilizing disposable screen-printed electrodes consisting of a platinum working electrode, a platinum counter electrode, and a silver reference electrode. Four different surface modifications, involving different combinations of Nafion (N) and enzyme (E) layers (N + E; N + E + N; N + N + E; N + N + E + N), were tested to identify the most effective configuration for a sensor that can be used for breath acetone detection. The sensor's essential characteristics, including linearity, sensitivity, reproducibility, and limit of detection, were thoroughly evaluated through a range of experiments spanning concentrations from 1 µM to 25 mM. Changes in acetone concentration were monitored by comparing currents readings at different acetone concentrations. The sensor exhibited high sensitivity, and a linear response to acetone concentration in both liquid and gas phases within the specified concentration range, with correlation coefficients ranging from 0.92 to 0.98. Furthermore, the sensor achieved a rapid response time of 30–50 s and an impressive detection limit as low as 0.03 µM. The results indicated that the sensor exhibited the best linearity, sensitivity, and limit of detection when four layers were employed (N + N + E + N).

## Introduction

Acetone, a metabolic byproduct resulting from fat breakdown, serves as a valuable marker for assessing fat metabolism and dietary adherence^1^. Previous research has linked breath acetone levels to weight loss via fat reduction^1,2^. Exercise leads to increased acetone levels due to glycogen depletion and greater reliance on fat for energy^1^. Monitoring daily breath acetone can reveal fluctuations in fat metabolism influenced by diet and exercise^1,3,4^. In healthy individuals, acetone is typically present in very low concentrations in breath, often ranging from baseline levels (close to 0 ppm) up to a few parts per million (ppm). The exact concentration can fluctuate throughout the day and may be influenced by factors such as fasting, exercise, and the composition of one's diet^2–5^.

During fasting or periods of low carbohydrate intake, such as during a ketogenic diet, acetone levels in breath can increase as the body metabolizes fat for energy, leading to the production of ketone bodies, including acetone. In individuals following a ketogenic diet or during fasting, acetone concentrations in breath may range from a few ppm to higher levels, depending on the degree of ketosis^6,7^.

In certain medical conditions such as diabetic ketoacidosis (DKA), where there is a significant increase in ketone production due to uncontrolled diabetes, acetone levels in breath can rise substantially. In severe cases of DKA, acetone concentrations in breath can exceed 100 ppm or even higher^7^.

Obese individuals exhibit higher acetone concentrations, with levels affected by factors like cardiorespiratory fitness^5^, diabetes (exceeding 1800 ppm in patients)^6^, rigorous physical activity^6^, and ketogenic diets (several hundred ppm)^7,8^. Elevated exhaled acetone has ties to adverse health outcomes and potential heart failure biomarker status. Consequently, measuring breath acetone and linking it to various diseases for diagnosis is essential.

Commercial acetone gas sensors operate between 50 and 5000 ppm, exceeding the physiological range of (1 ppm in healthy non‐dieting subjects to 1250 ppm in diabetic ketoacidosis)^9^. Therefore, developing techniques for physiological-range breath acetone measurement becomes imperative. Common methods within this range include gas chromatography-mass spectrometry (GC–MS), ion mobility spectrometry-mass spectrometry (IMS-MS), and others^10–16^. However, these techniques are costly and lack portability for real-time monitoring.

Alternatively, accessible methods include low-frequency a.c. response sensors, optical-fiber sensors, and colorimetric sensors^17–20^. Recent trends favor miniaturized sensors, including electrochemical gas sensors for portable, cost-effective diabetes monitoring^21–38^. Electrochemical sensors, known for their simplicity, affordability, and selectivity, could potentially be used in handheld, disposable diabetes monitoring devices^39,40^. These sensors for measuring acetone can be categorized as non-enzymatic or enzymatic.

Non-enzymatic breath acetone sensors necessitate specific electrolytes, like sulfuric acid (H_2_SO_4_) or sodium tartrate (Na_2_C_4_H_4_O_6_), to initiate acetone reactions. These reactions generate an electrochemically active product, quantified via redox reactions, commonly using amperometry or cyclic voltammetry (CV), with current proportionate to breath acetone concentration^22,28,32^.

Wang et al.^22^ presented a novel non-enzymatic acetone sensor using a lead foil electrode in a 0.1 M sodium tartrate solution. Amperometry revealed a strong linear relationship between response current and acetone concentration (50–250) ppm. Motsegood et al.^28^ developed a non-enzymatic sensor using cyclic voltammetry, capable of measuring breath acetone concentrations (1 μM to 10 mM) through the electrolysis of adsorbed acetone.

Obeidat et al.^32^ developed a non-enzymatic sensor for liquid and vapor acetone using disposable screen-printed electrodes in H_2_SO_4_ solution. Their sensor displayed a strong linear response (1 µM to 10 mM).

Enzymatic acetone sensors rely on an enzyme system generating H_2_O_2_ when exposed to acetone. Detection occurs through amperometry or cyclic voltammetry, with current proportionate to acetone concentration^21,23,24^. Landini et al.^23,24^ designed a compact enzymatic sensor for breath acetone measurement in dieting individuals, with a range of 0.2 to 10 ppm.

Our study introduces a novel enzymatic sensor using solid-state electrolytes, disposable screen-printed electrodes, and cyclic voltammetry and amperometry techniques. Experiments included four surface modifications (N + E, N + E + N, N + N + E, N + N + E + N) to optimize detecting of acetone for diabetes monitoring. Key characteristics, such as linearity, sensitivity, reproducibility, and limit of detection (LOD), were thoroughly assessed across a wide concentration range (1 µM to 25mM).

## Materials and methods.

### Sensor electrodes

**Figure 1:**
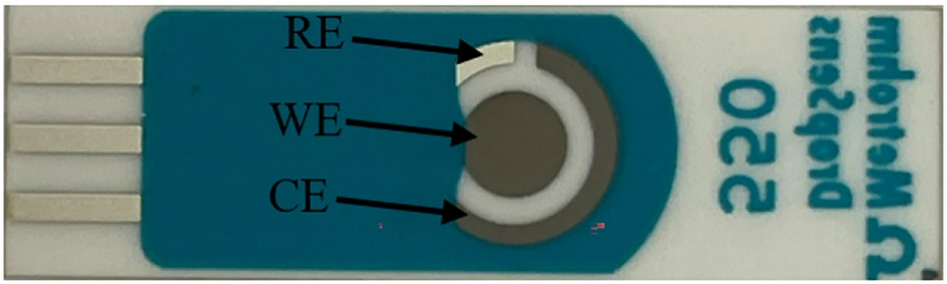
DropSens screen printed electrodes.

All experiments employed DS550 disposable screen-printed electrodes (DropSens, Llanera, Spain). As outlined on the DropSens website, these electrodes were fabricated on a ceramic substrate measuring 33 mm in length, 10 mm in width, and 0.5 mm in height. The electrochemical setup comprised a circular platinum (Pt) working electrode (WE) with a diameter of 4 mm, a curved Pt counter electrode (CE), a small curved silver (Ag) reference electrode (RE), and a silver electrical contact, as depicted in Fig. 1. The selection of a Pt working electrode was based on its exceptional stability and its capacity to exhibit excellent sensitivity towards electrochemical reactions^29,41^.

### Reagents

Pure acetone (Sigma Aldrich, USA) was employed in the experiments. To prepare the acetone samples, 250 mL flasks were utilized, while a 2 mL flask was employed to prepare the enzyme. Vapor acetone samples were introduced using a 50 mL syringe, while liquid acetone samples were injected using a micropipette (Sigma Aldrich, USA) with a range of (10–100) μL. Nafion and enzyme layers were injected using a Hamilton microsyringe (Sigma Aldrich, USA) with a volume range of (0.1–10) μL. Deionized (DI) water obtained from a Milli-Q plus water filtration system was used both to dilute acetone solutions and to rinse the electrode surface between measurements. A 5% w/w Nafion perfluorinated resin (Sigma Aldrich, USA, product number: 527084, Nafion™ perfluorinated resin solution 5 wt% in mixture of lower aliphatic alcohols and water, contains 45% water) was used as a solid state electrolyte in all experiments. Additionally, potassium phosphate powder (Sigma Aldrich, USA, MW: 136.09, product number: 7778-77-0), L-lactate powder (Sigma Aldrich, USA, MW: 218.22, EC No.: 248-953-3), secondary alcohol dehydrogenase (S-ADH) (Sigma Aldrich, USA, EC No.:232-870-4, product number: A3263, powder, ≥ 300 units/mg protein, mol wt ~ 141,000 (four subunits)), lactate dehydrogenase (LDH) from hog muscle (Sigma Aldrich, USA, Enzyme Commission Number:1.1.1.27 (BRENDA, IUBMB), Product Number: 0107085001, ~ 550 units/mg protein (at 25 °C (1000 U/mg at 37 °C) with pyruvate as the substrate.), nicotinamide adenine dinucleotides (NADH) cofactor (Sigma Aldrich, USA, MW: 663.43, EC No.: 200-184-4), and pyruvate oxidase (PO) from Aerococcus sp (Sigma Aldrich, USA, EC No.: 232-644-5, lyophilized powder, ≥ 35 units/mg protein (biuret)) were used in the preparation of the enzyme.

### Enzyme preparation

The enzyme solution was prepared by following a modified version of the procedure described in^21^. A 1 mL of a 50 mM potassium phosphate buffer was placed in a 2 mL vessel. Subsequently, an appropriate quantity of lactate was introduced into the buffer to achieve a final lactate concentration of 10 mM. Gentle mixing with a stirring rod was employed to ensure thorough blending. Subsequently, 3 µg of purified S-ADH was added and stirred until a uniform mixture was attained. NADH was then added to the buffer to reach a concentration of 50 µM. Following this, 20 units of LDH and 2 units of PO were incorporated. Mixing was continued until a homogeneous mixture was achieved, and the pH level was monitored to verify that it remained around 6.2.

### Sensor surface modification

The sensor surface underwent modification through the sequential addition of Nafion and enzyme layers. Each Nafion layer was formed by applying a 0.2 μL of Nafion solution onto the working electrode (WE) surface, allowing it to air-dry for 30 min at room temperature, resulting in a thin Nafion layer. Likewise, each enzyme layer was established by applying a 0.8 μL of the prepared enzyme solution to cover the WE surface, followed by a 20-min air-drying period at room temperature. This process resulted in the creation of four distinct surface modifications, characterized by various combinations of Nafion and enzyme layers, specifically: N + E, N + E + N, N + N + E, and N + N + E + N. The electrodes that were prepared in this manner were stored in a refrigerator at 4°C until they were ready for use.

### Enzyme system

**Figure 2:**
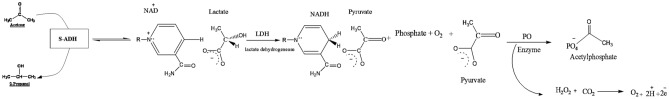
The mechanism of enzymatic breath acetone detection.

The enzyme mixture comprised S-ADH, lactate dehydrogenase (LDH), and pyruvate oxidase (PO) when examined in a laboratory setting^21,23^. In the context of detecting breath acetone (propanone), a three-enzyme process is employed^23^. This process begins by utilizing S-ADH, along with the NADH cofactor, in a catalytic reaction as the initial stage. In this step, acetone from the breath sample undergoes reduction, transforming into 2-propanol in the presence of S-ADH, which contains a zinc metalloenzyme. Simultaneously, NADH is converted into NAD + . Zinc plays a role as a Lewis acid, serving as an electrophilic center to facilitate the transfer of hydride ions. The presence of NAD + initiates the conversion of lactate to pyruvate. In the final stage of this process, pyruvate is converted into acetyl phosphate, hydrogen peroxide (H_2_O_2_), and carbon dioxide (CO_2_) in the presence of PO, phosphate, and O_2_. Consequently, oxidation of the produced H_2_O_2_ will begin by applying the voltage potential required for the oxidation/reduction reaction to occur on the Pt WE.^42^, the mechanism of enzymatic breath acetone detection is illustrated in Fig. 2.

### Electrochemical instrumentation

The PalmSens4 potentiostat (PalmSens, The Netherlands) was used to perform cyclic voltammetry (CV) and amperometry in all experiments. The potentiostat was connected to a PC that has PSTrace software to display and save the amperometry and CV data to generate the calibration curves.

### Acetone sample procedure

#### Liquid sample preparation

The liquid acetone samples were prepared by diluting a 13.5 M stock solution of acetone with DI water using a standard dilution method^43^. To achieve a concentration of 50 mM acetone, the stock solution was mixed with DI water in a 50 mL glass flask, sealed with a Parafilm and a septum for stability. Subsequently, the resulting 50 mM solution was used as a basis for preparing various concentrations ranging from 1 µM to 25 mM through further dilution.

### Gas (vapor) sample preparation

The gas samples were prepared at room temperature using the identical approach as that utilized in our previous research^32^. A 25 mL acetone solutions was mixed with varying concentrations in a 50 mL glass flask, sealing it tightly with Parafilm and a septum. These samples were allowed to sit undisturbed for 30 min to facilitate the generation of vapor samples and ensure their even distribution within the flask, mimicking the characteristics of breath samples. Due to the direct correlation between the amount of acetone vapor in the headspace and the concentration of the solution, we reported the calibration measurements of the vapor phase samples in terms of acetone concentration within the aqueous solution. To enhance measurement stability and minimize errors, the closed glass system designed in our previous published work^32^ was employed for gas measurements. The glass container features a side for electrode insertion, an air outlet for evacuating any gas prior to measurements, and a sample inlet for introducing the acetone sample. This system reduces acetone exchange with the surrounding air during measurements, thereby ensuring improved stability and reduced inaccuracies.

### The measurement setup

The experiments were conducted in the Research Lab for Designing Sensors and Systems (Electronics Engineering Department, Yarmouk University). Cyclic voltammetry and amperometry were employed in this work to measure acetone concentrations across a range of 1 µM to 25 mM. Cyclic voltammetry was used to determine the H_2_O_2_ oxidation/reduction potential as well as the acetone concentration within a specific range by scanning the voltage from (− 1.3 to 1.3) V. Subsequently, the voltage obtained from cyclic voltammetry was applied in amperometry measurements conducted under varying modifications and concentrations. The results obtained from amperometry were utilized to establish calibration curves for the sensor across different surface modifications.

To create the acetone solutions, we followed the steps detailed in "Acetone sample procedure" section. Throughout the measurements, we set the voltage to match the potential identified via cyclic voltammetry. The measurements were conducted sequentially, starting from lower concentrations and progressing to higher ones, encompassing acetone concentrations from 1 µM to 25 mM. As a reference solution containing zero acetone, DI water was used. DI water was also employed for rinsing the electrode between measurements to ensure a clean surface before introducing new sample.

#### Liquid sample experiments

##### Cyclic voltammetry

Prior to each experiment, the electrodes underwent a cleansing process using DI water and were air-dried at room temperature. The liquid samples were prepared according to the procedure outlined in the Materials and Methods section. To establish a baseline measurement, 50 µL of DI water was introduced onto the electrodes, and a scanning procedure was carried out, spanning from − 1.3 to + 1.3 V at a rate of 100 mV/s, repeated for 3 cycles. For each liquid sample within the concentration range of 1 µM to 25 mM, a 50 µL volume was directly applied onto the electrodes surface, followed by the same scanning procedure between − 1.3 V and + 1.3 V at 100 mV/s for 3 cycles. Accordingly, this procedure was repeated for all sensors with different surface modifications.

##### Amperometry

Before each experiment, the electrodes underwent a cleaning procedure using DI water and were then left to air-dry at room temperature. The liquid samples were prepared following the steps outlined in the Materials and Methods section. To establish a baseline measurement, A 50 µL of DI water was added onto the electrodes. The voltage was determined based on the results obtained from the cyclic voltammetry for each specific surface modification, and amperometry was conducted for 210 s to record the current value corresponding to a zero acetone concentration.

For each liquid sample falling within the concentration range of 1 µM to 25 mM, a 50 µL volume was directly applied onto the electrodes surface. Subsequently, the same measurement procedure was repeated, utilizing the same voltage and time duration, to measure the current value corresponding to each specific acetone concentration. This process was replicated for all sensors with differing surface modifications.

#### Vapor sample experiments

The glass system was employed to analyze vapor samples prepared according to procedures explained in the materials and methods section. To create a secure, airtight setup, the system was hermetically sealed, and a vacuum pump evacuated any remaining air until pressure stabilized. The voltages determined from cyclic voltammetry (CV) was used for liquid samples with different surface modifications.

In each experiment, a baseline current was established through amperometry after injecting air collected from DI water's surface. Subsequently, current values were recorded for different acetone concentrations. The vapor sample extracted from the head space above the acetone liquid was collected using a 50 mL syringe, which was filled with 25 mL of acetone gas and manually injected through the gas inlet. A timer was utilized to maintain a consistent flow rate of 2 mL/s for each injected sample. To ensure precise current readings, amperometric measurements were conducted 2 min after the introduction of acetone vapor, ensuring its diffusion to the electrodes.

After each measurement, DI water was used to clean the electrodes, and the vacuum pump removed any remaining air between readings. This process was repeated three times to eliminate any residual acetone from prior measurements. This procedure was applied to all sensors with different surface modifications.

## Results and discussion

### Sensor cyclic voltammetry results

**Figure 3:**
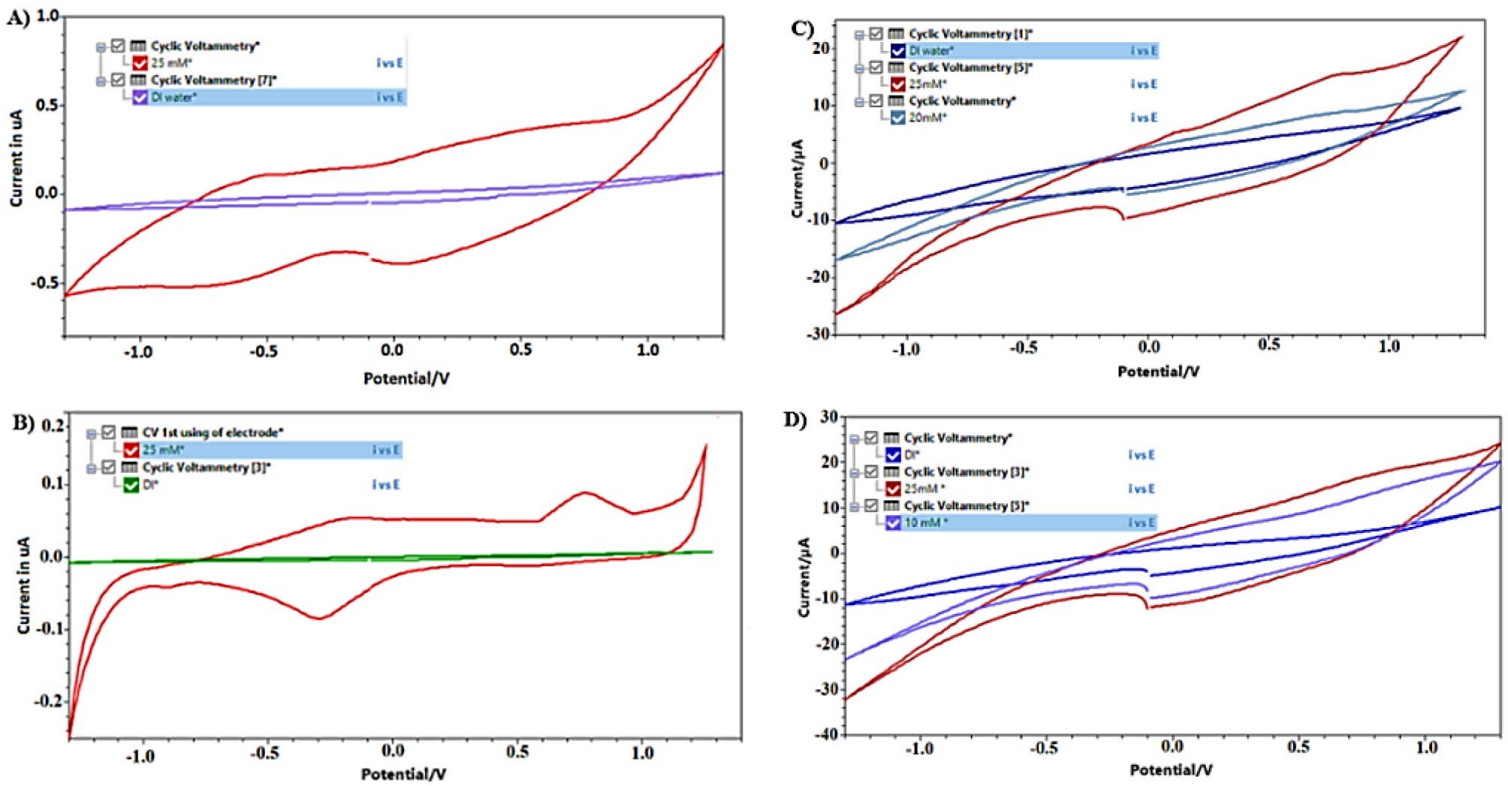
CV results for different surface modifications: (**A**) N + E. (**B**) N + N + E + N. (**C**) N + N + E. (**D**) N + E + N.

The cyclic voltammetry was done to measure the oxidation current at each concentration, the results have shown the change in the oxidation peak compared to the zero reading after adding the acetone sample and the oxidation current increases with the increase in acetone concentration. Figure 3 shows an example of the cyclic voltammetry results collected for all types of surface modifications. The blank DI water represent the baseline measurement (zero acetone level). The acetone oxidation current was measured at 700 mV for the sensor covered with N + E, and it was measured at 900 mV for all three and four layers’ systems (i.e. N + E + E; N + E + N; N + N + E + N). The potential determination relies on the current increase as concentrations increase at the same potential. While there are additional peaks in the figures, they do not correspond to acetone since they do not exhibit the correct trend with varying concentrations.

The oxidation potential of H_2_O_2_ in our study differed from the voltage values reported in existing literature^42^ for three specific reasons. Firstly, the variation is due to the difference in the electrodes’ shape and surface area. Secondly, while the conventional reference electrode typically consists of silver/silver chloride, a reference electrode composed solely of silver was employed. Lastly, the working electrode (WE) featured distinct layers that could potentially alter its properties. The peak where acetone can be measured is still clear in all figures and this can be verified by observing the rise in the current peak corresponding to an increase in acetone concentration at the same potential. However, some IR drop appears in Figs. (5C and 5D), this may be attributed to occasional human errors in the injection process of Nafion and/or enzyme, resulting in uneven layer distribution on the surface. This unevenness could lead to uncompensated resistance between the reference electrode tip and the working electrode surface. Consequently, there is a reduction in the effective potential applied to the electrochemical double layer^44^.

### Sensor calibration results

#### Liquid acetone samples

**Figure 4:**
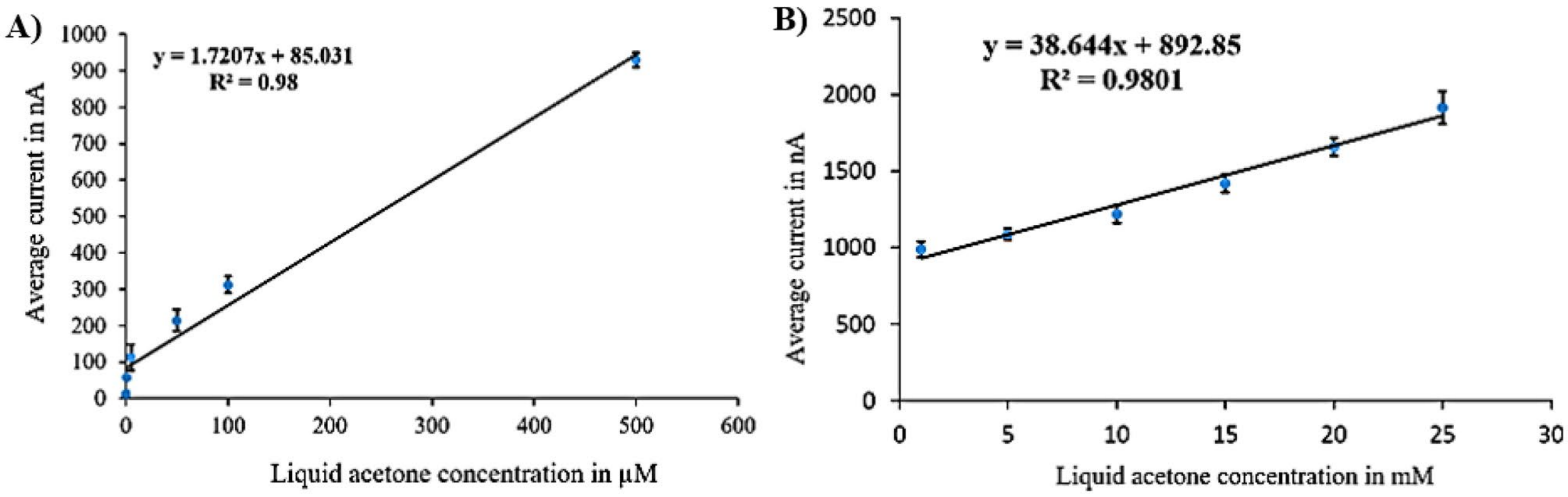
Calibration curves for liquid acetone in case of N + N + E + N: (**A**) low concentration. (**B**) high concentration.

**Figure 5:**
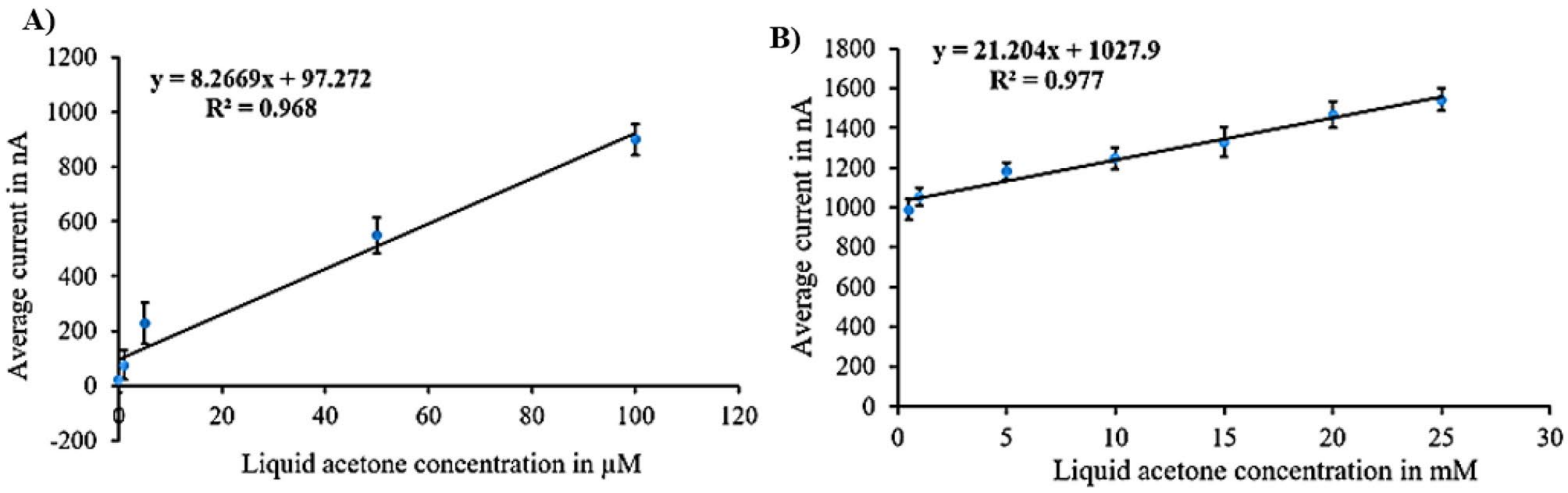
Calibration curves for liquid acetone in case of N + N + E: (**A**) low concentration. (**B**) high concentration.

**Figure 6:**
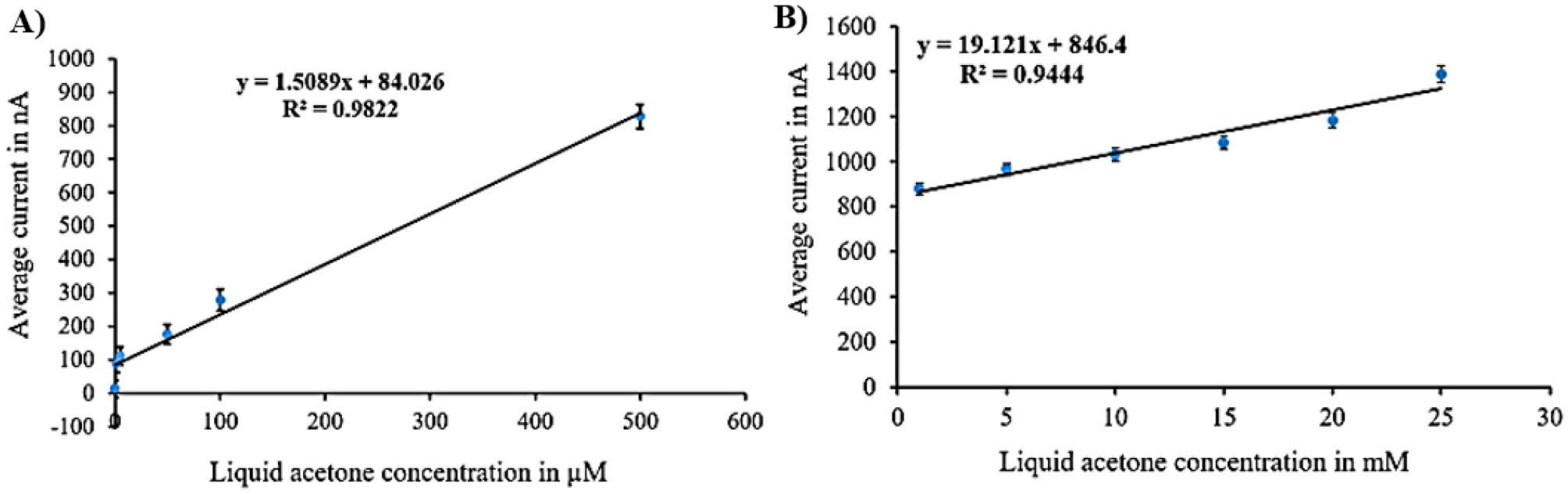
Calibration curves for liquid acetone in case of N + E + N: (**A**) low concentration. (**B**) high concentration.

**Figure 7:**
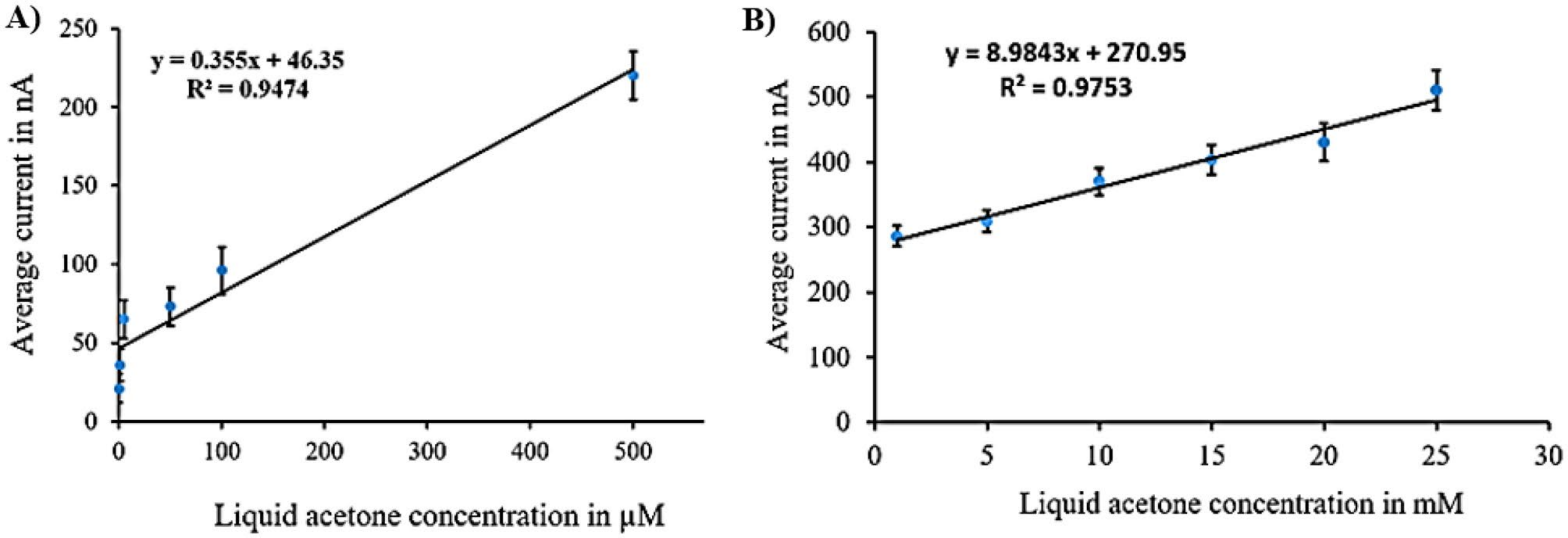
Calibration curves for liquid acetone in case of N + E: (**A**) low concentration. (**B**) high concentration.

The sensor calibration curves (Figs. 4, 5, 6 and 7) were derived from current values corresponding to various acetone concentrations ranging from 1 µM to 25 mM, collected through amperometry at voltages determined by cyclic voltammetry results. The current values were recorded during the period when the signal stabilizes, typically within a timeframe of 3–5 s. We compute the average of these stable readings within a defined range. The calibration curves illustrate the average readings collected from six electrodes under uniform conditions. For each surface modification, two calibration curves were established, one for lower concentrations (1 µM to 500 µM) and another for higher concentrations (1 mM to 25 mM).

The calibration curves for the (N + N + E + N) surface modification are illustrated in Fig. 4. Figure 4A depicts the calibration curve for the lower concentration range, with average currents from 13 nA at zero acetone level to 930 nA at 500 µM, showing a sensitivity of 1.721 nA/µM and a correlation coefficient of 0.98 and the variance error is (σ^2^ = 123). Figure 4B displays the calibration curve for the higher concentration range, with average currents from 988.75 to 1915 nA, indicating a sensitivity of 38.64 nA/mM and a correlation coefficient of 0.98 and the variance error is (σ^2^ = 134).

Figure 5 illustrates the calibration curves for the (N + N + E) surface modification. Figure 5A represents the calibration curve for the lower concentration range, with average currents from 20 nA at zero acetone level to 900 nA at 500 µM level, revealing a sensitivity of 8.267 nA/µM and a correlation coefficient of 0.968 and the variance error is (${\sigma }^{2}=81$). Figure 5B showcases the calibration curve for the higher concentration range, with average currents from 990 to 1542.2 nA, displaying a sensitivity of 21.2 nA/mM and a correlation coefficient of 0.977 and the variance error is (${\sigma }^{2}=124$).

The calibration curves for the (N + E + N) surface modification are shown in Fig. 6. Figure 6A illustrates the calibration curve for the lower concentration range, with average currents from 12 nA at zero acetone level to 828 nA at 500 µM, indicating a sensitivity of 1.509 nA/µM and a correlation coefficient of 0.982 and the variance error is (${\sigma }^{2}=36.5$). Figure 6B displays the calibration curve for the higher concentration range, with average currents from 879 to 1386.2 nA, showing a sensitivity of 19.12 nA/mM and a correlation coefficient of 0.944 and the variance error is (${\sigma }^{2}=13.4$).

Figure 7 shows the calibration curves for the (N + E) surface modification. Figure 7A shows the calibration curve for the lower concentration range, with average currents from 21 nA at zero acetone level to 220 nA at 500 µM, revealing a sensitivity of 0.355 nA/µM and a correlation coefficient of 0.947 and the variance error is (${\sigma }^{2}=4.8$). Figure 7B illustrates the calibration curve for the higher concentration range, with average currents from 285.75 to 511 nA, displaying a sensitivity of 8.98 nA/mM and a correlation coefficient of 0.975 and the variance error is (${\sigma }^{2}=34$).

**Figure 8:**
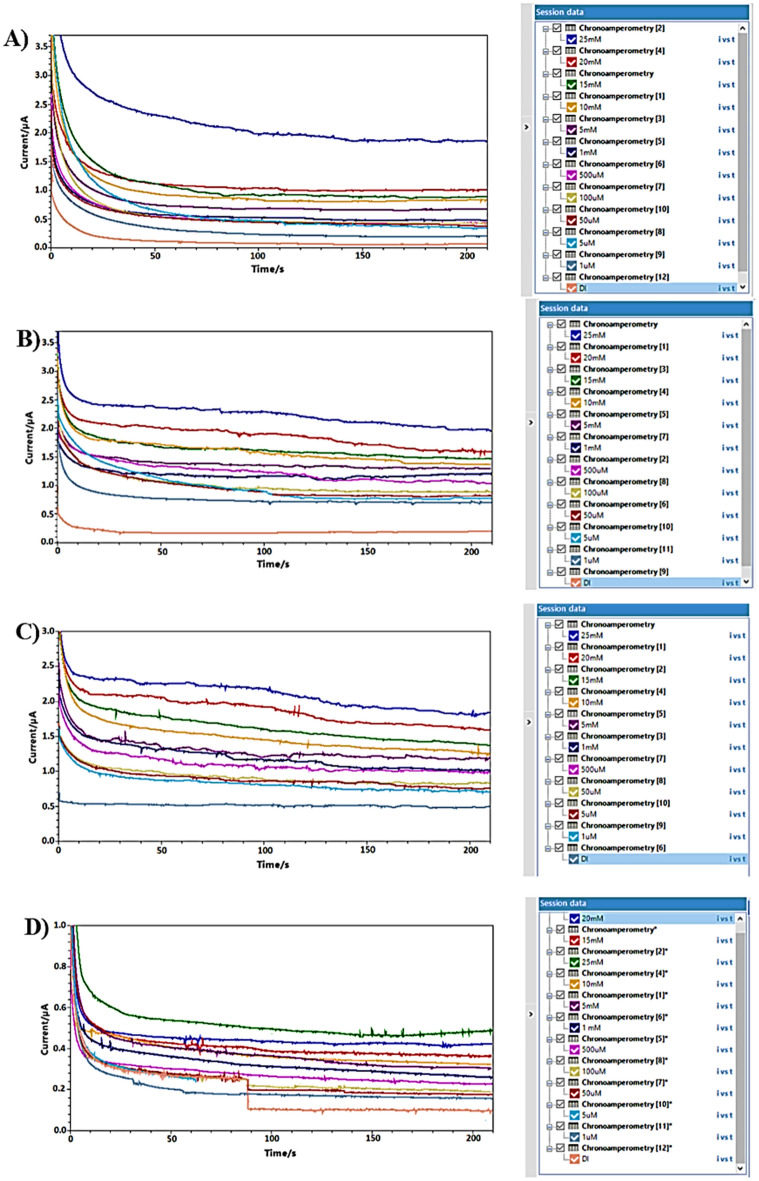
Example of amperometry results of liquid samples across concentration range of (1 µM to 25 mM) at different surface modifications of: (**A**) N + N + E + N. (**B**) N + N + E. (**C**) N + E + N. (**D**) N + E.

The calibration curves clearly indicate that the (N + N + E + N) configuration provided the best combination of linearity and sensitivity across both concentration ranges. Specifically, in the low concentration range, the (N + N + E) configuration exhibited the highest sensitivity among all tested setups. Raw data from amperometry under all surface modifications is depicted in Fig. 8.

### Vapor acetone samples

**Figure 9:**
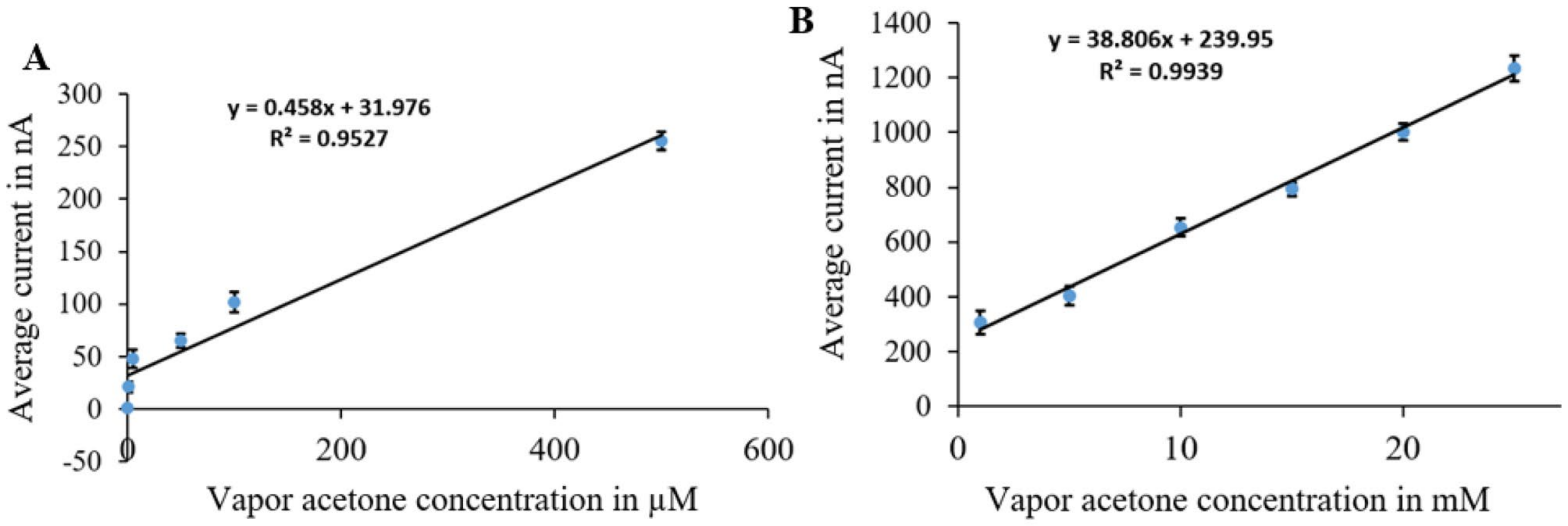
Calibration curves for vapor acetone in case of N + N + E + N: (**A**) low concentration. (**B**) high concentration.

**Figure 10:**
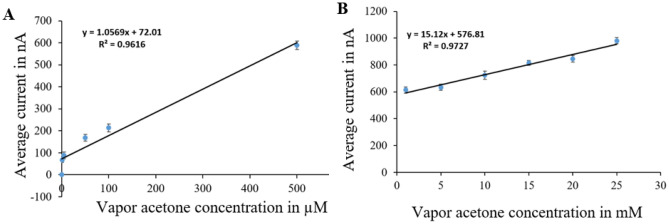
Calibration curves for vapor acetone in case of N + N + E: (**A**) low concentration. (**B**) high concentration.

**Figure 11:**
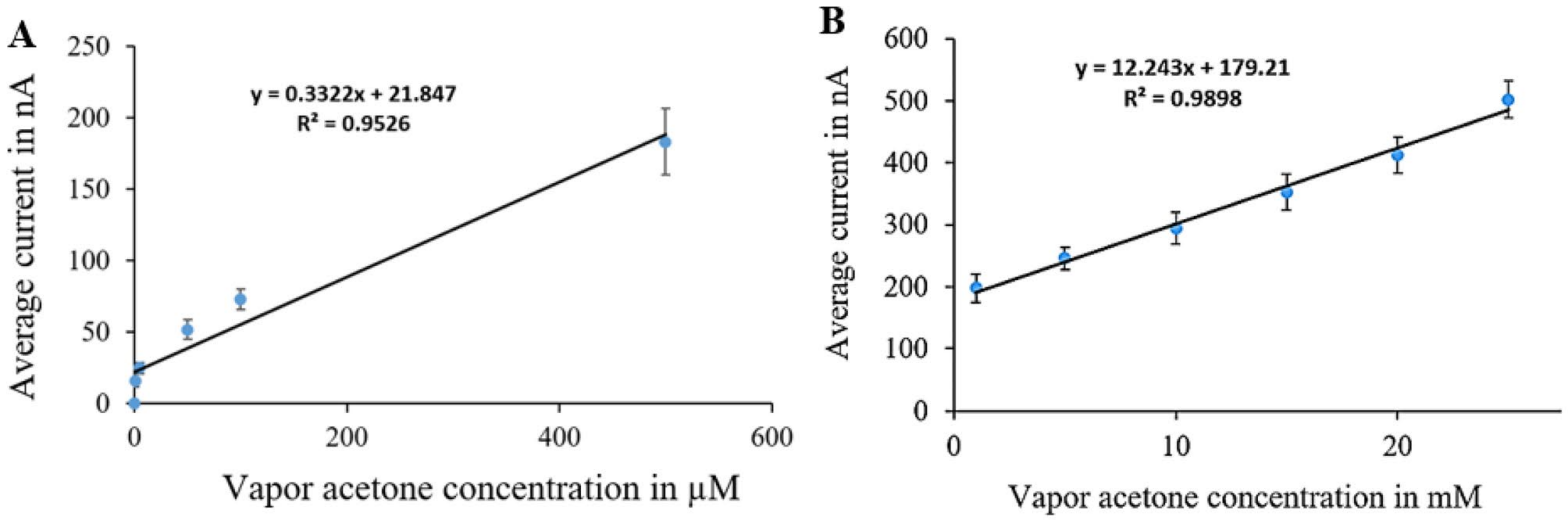
Calibration curves for vapor acetone in case of N + E + N: (**A**) low concentration. (**B**) high concentration.

**Figure 12:**
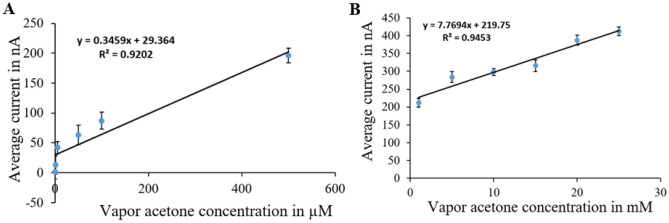
Calibration curves for vapor acetone in case of N + E: (**A**) low concentration. (**B**) high concentration.

The sensor calibration curves (Figs. 9, 10, 11 and 12) relate current values to acetone concentrations ranging from 1 µM to 25 mM, determined through amperometry with voltages from cyclic voltammetry results. For each surface modification, two calibration curves were established: one for concentrations from 1 µM to 500 µM and another for concentrations from 1 to 25 mM.

In Fig. 9, the calibration curves for the (N + N + E + N) surface modification are depicted. Figure 9A shows the curve for the lower concentration range, with average currents from 1.3 nA at zero acetone level to 255 nA at 500 µM, sensitivity of 0.458 nA/µM, and a correlation coefficient of 0.953 and the variance error is (${\sigma }^{2}=7.5$). Figure 9B displays the curve for the higher concentration range, with average currents from 304 to 1234 nA, sensitivity of 38.806 nA/mM, and a correlation coefficient of 0.99 and the variance error is (${\sigma }^{2}=52$)..

In Fig. 10, calibration curves for the (N + N + E) surface modification are presented. Figure 10A represents the curve for the lower concentration range, with average currents from 0.4 nA at zero acetone level to 589 nA at 500 µM, sensitivity of 1.057 nA/µM, and a correlation coefficient of 0.962 and the variance error is (${\sigma }^{2}=37$). Figure 10B shows the curve for the higher concentration range, with average currents from 613 to 980 nA, sensitivity of 15.12 nA/mM, and a correlation coefficient of 0.973 and the variance error is (${\sigma }^{2}=17.6$).

Figure 11 displays calibration curves for the (N + E + N) surface modification. Figure 11A illustrates the curve for the lower concentration range, with average currents from 0 nA at zero acetone level to 183 nA at 500 µM, sensitivity of 0.332 nA/µM, and a correlation coefficient of 0.953 and the variance error is (${\sigma }^{2}=54$).. Figure 11B demonstrates the curve for the higher concentration range, with average currents from 198 to 502 nA, sensitivity of 12.24 nA/mM, and a correlation coefficient of 0.989 and the variance error is (${\sigma }^{2}=17.5$).

Figure 12 presents calibration curves for the (N + E) surface modification. Figure 12A showcases the curve for the lower concentration range, with average currents ranging from 2.1 nA at zero acetone level to 196 nA at 500 µM, sensitivity of 0.346 nA/µM, and a correlation coefficient of 0.92 and the variance error is (${\sigma }^{2}=22$). Figure 12B displays the curve for the higher concentration range, with average currents from 212 to 412 nA, sensitivity of 7.77 nA/mM, and a correlation coefficient of 0.945 and the variance error is (${\sigma }^{2}=5.2$).

**Figure 13:**
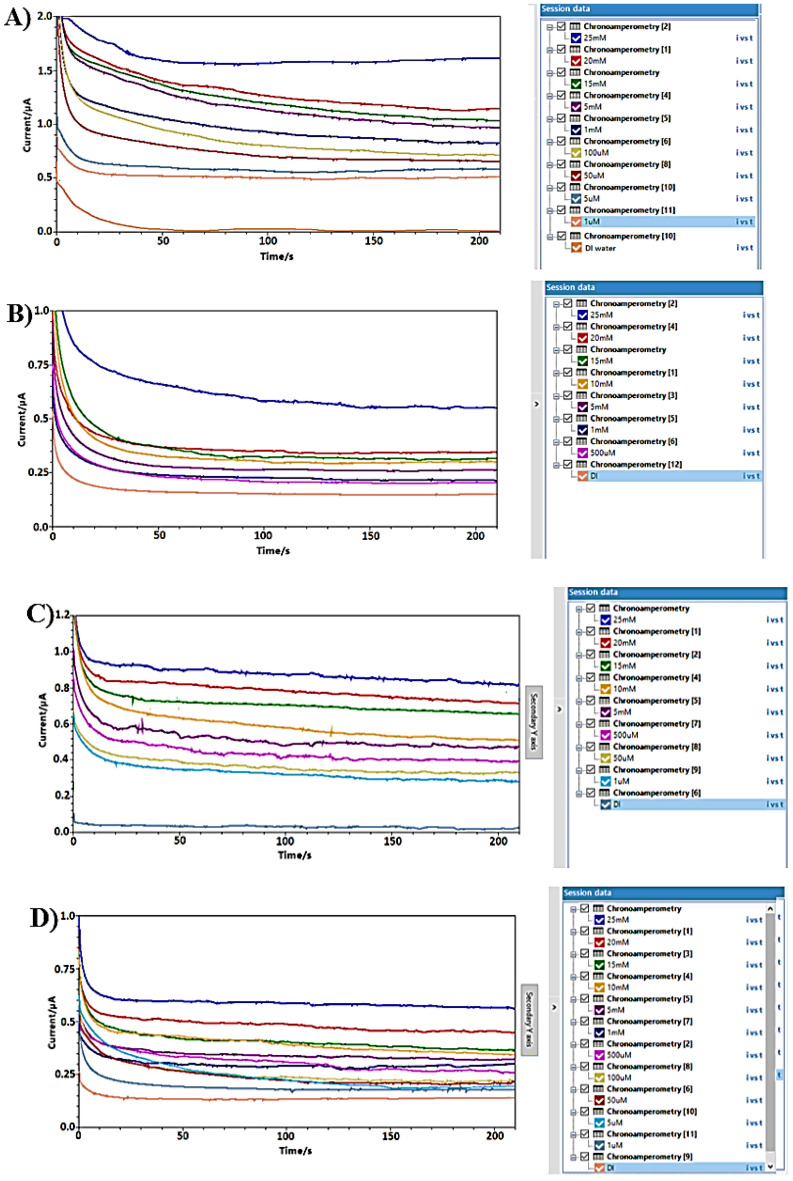
Example of amperometry results of vapor samples across concentration range of (1 µM to 25 mM) at different surface modifications of: (**A**) N + N + E + N. (**B**) N + N + E. (**C**) N + E + N, (**D**) N + E.

Calibration curves from vapor samples yielded similar results to liquid samples. The (N + N + E + N) configuration exhibited the best linearity and sensitivity across both concentration ranges. Additionally, the (N + N + E) configuration showed the highest sensitivity in the low concentration range. An example of raw data from amperometry under all surface modifications is provided in Fig. 13.

### Summary of the results

Incorporating all surface modifications, the sensor accurately measured a wide range of liquid and vapor acetone concentrations from 1 μM to 25 mM. Taking into account Henry’s law^45^ and considering a Henry constant of 0.27 mol(m^3^Pa) or 27.3578 M/atm for acetone, this span extends from 0.0366 parts per million (ppm) to 913.8 ppm. The sensor maintained excellent linearity and high sensitivity, determined by the calibration curve's slope. The sensor's current output displayed a linear relationship with increasing acetone concentration in both low and high ranges. To evaluate measurement reliability and reproducibility, error bars represented the standard deviation of six calibration data points from six identical electrodes under the same surface modifications for liquid sample experiments. For vapor sample experiments, error bars represented the standard deviation of four calibration data points from four identical electrodes under identical surface modifications. Results indicated the sensor's consistent response and reproducibility to changes in acetone concentration across all surface modifications. Variations in measurement outcomes could be attributed to factors like injection speed and direction of acetone samples, variations in enzyme and Nafion layer thickness, rapid acetone evaporation in an open-air environment, potential differences in acetone sample concentrations, potential electrochemical interference between electrodes, and possible retention of acetone samples on chamber walls during vapor sample testing. A summery table for all results is shown in Table 1 below.

**Table 1:** A summary table for all results.

Sample state	Type of layers	Concentration range	Sensitivity	Linearity (R^2^)
Gas	N + N + E + N	Low: (1–500) µM (0.0366–18.3)ppm	0.458 nA/µM	0.953
		High: (1–25) mM (36.55–913.8)ppm	38.806 nA/mM	0.99
	N + N + E	Low: (1–500) µM (0.0366–18.3)ppm	1.057 nA/µM	0.962
		High: (1–25) mM (36.55–913.8)ppm	15.12 nA/mM	0.973
	N + E + N	Low: (1–500) µM (0.0366–18.3)ppm	0.332 nA/µM	0.953
		High: (1–25) mM (36.55–913.8)ppm	12.24 nA/mM	0.989
	N + E	Low: (1–500) µM (0.0366–18.3)ppm	0.346 nA/µM	0.92
		High: (1–25) mM (36.55–913.8)ppm	7.77 nA/mM	0.945
Liquid	N + N + E + N	Low: (1–500) µM	1.721 nA/µM	0.98
		High: (1–25) mM	38.64 nA/mM	0.98
	N + N + E	Low: (1–500) µM	8.267 nA/µM	0.968
		High: (1–25) mM	21.2 nA/mM	0.977
	N + E + N	Low: (1–500) µM	1.509 nA/µM	0.982
		High: (1–25) mM	19.12 nA/mM	0.944
	N + E	Low: (1–500) µM	0.355 nA/µM	0.947
		High: (1–25) mM	8.98 nA/mM	0.975

### Limit of detection (LOD)

**Figure 14:**
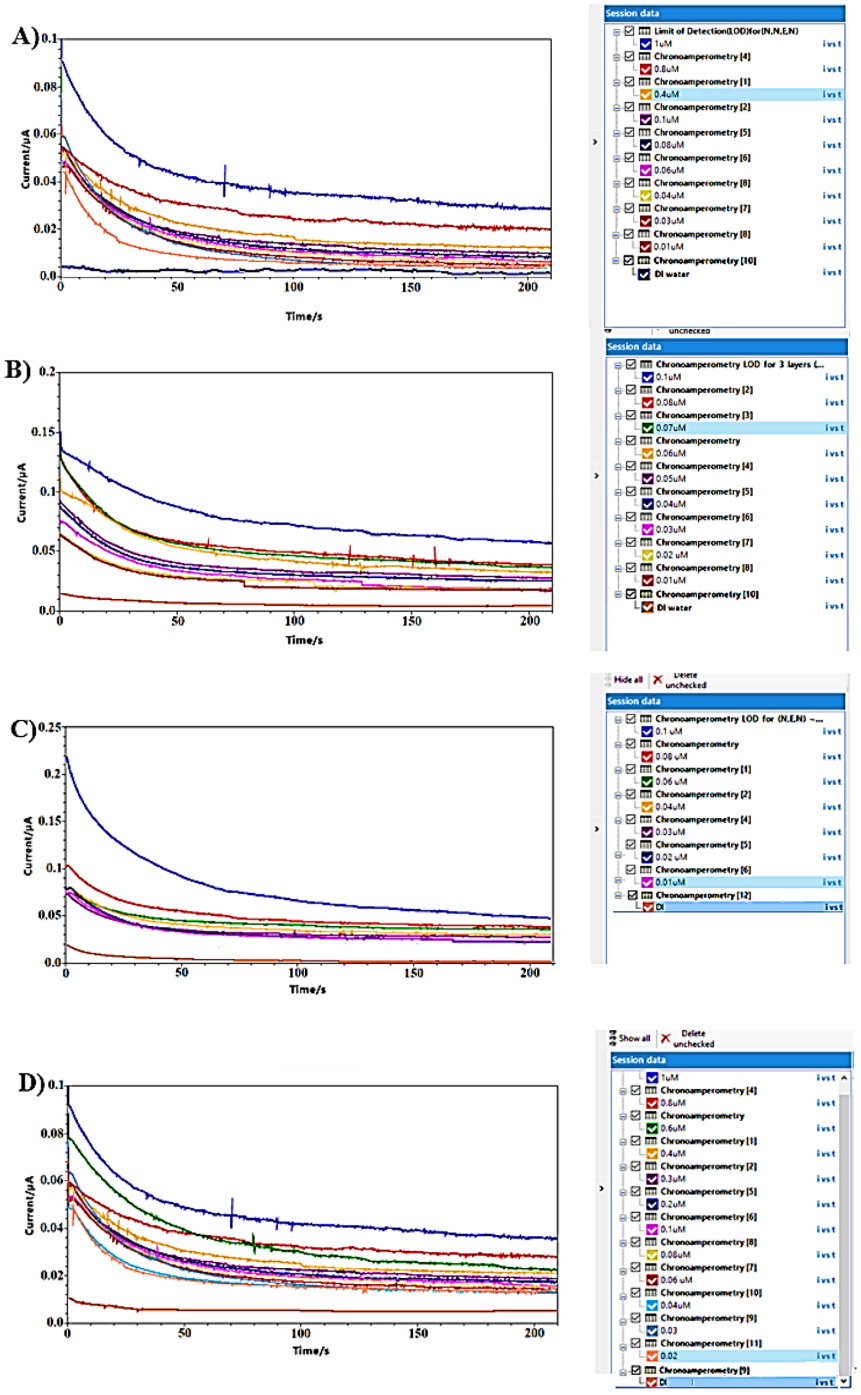
Example of amperometry results for measuring the LOD at different surface modifications of: (**A**) N + N + E + N. (**B**) N + N + E. (**C**) N + E + N. (**D**) N + E.

The limit of detection (LOD) was determined by diluting acetone samples to extremely low concentration levels, selecting the minimum concentration detectable from a sample with zero acetone content. Dilution was conducted for liquid acetone within a concentration range below 0.1 μM, down to 0.01 μM. The LOD was measured based on a signal response to low concentration compared to the noise level in a zero acetone sample considering a signal to noise ratio of 3:1. As depicted in Fig. 14, the LOD for N + N + E + N, N + N + E, and N + E + N was approximately 0.03 μM, while it reached 0.04 μM for the N + E surface modification. This demonstrates the exceptional sensitivity of our sensor in detecting minute concentration changes, making it a promising choice for acetone detection in the human range, particularly for diabetes monitoring.

### Limit of quantification (LOQ)

The limit of quantification (LOQ) for the acetone sensor was determined across all conditions by applying a criterion of a signal-to-noise (S/N) ratio of 10:1. This means that the LOQ was established with the condition that the signal must surpass the noise by at least 10 times for dependable quantification. Given that the limit of detection (LOD) has already been determined, the LOQ was derived from the LOD using a commonly employed multiplier denoted as "k." This formula is expressed as LOQ = k * LOD^46^. While some methodologies opt for larger multipliers like 5 or 10 to ensure heightened reliability in quantification, in our study, we adopted k = 3, a frequently utilized value. Consequently, the LOQ for N + N + E + N, N + N + E, and N + E + N was estimated to be around 0.09 μM, whereas for the N + E surface modification, it was slightly higher at 0.12 μM.

### Comparison with the state of art

While various breath acetone measurement sensors have been explored, such as semiconductor-based, chemoresistive, colorimetric, and optical sensors, there's a noticeable lack of research focused on electrochemical techniques like cyclic voltammetry and amperometry^19,20,31,40,47–50^. Furthermore, advancements in this area have been limited. Few researchers have delved into enzyme-based acetone sensors, with some following a patent issuance in this domain and the creation of a handheld breath acetone device by Allen et al.^21,23–25,27^.

Our work presents the development of an innovative enzymatic acetone sensor employing solid-state electrolytes and disposable screen-printed platinum electrodes. This sensor offers compatibility for integration with other sensing technologies and simplifies the construction of portable acetone measurement devices, holding promise across various applications, particularly in the medical field.

Compared to recently published literature, our sensors exhibit superior linearity, sensitivity, limit of detection (LOD), and reproducibility^19,20,31,32,51^. When compared to similar enzymatic sensors in previous research, our work stands out with advanced surface characterization, solid-state electrolyte implementation, and improved performance in terms of linearity, sensitivity, and LOD across all surface modifications. The measurement range of our sensor surpasses that of Landini et al.^23,24^,displaying significantly higher sensitivity, a lower LOD, and better linearity for specific surface modifications. Moreover, their sensor's calibration curves offer only a single data point, lacking information on reproducibility or the type of electrolyte used. The comparison of this work with the state of art is summarized in Table 2.

**Table 2:** The comparison of this work with the state of art.

References	Sensing method	Range	sensitivity	Linearity (R^2^)	LOD
^51^	Chemical sensor (polypyrrole(PPy)–Silver/silver chloride (Ag/AgCl) ternary nanocomposites (NCs)	(1–30) ppm	20%	Good (no information about R^2^)	1 ppm
^50^	Optical	(0.5–150) mM	No information	Good (no information about R^2^)	0.5 mM
^23,24^	Enzymatic sensor (amperometry)	(0.2–24) ppm	Good (number is not included)	> 0.95	No information
^28^	Cyclic voltammetry	1µM to 10 mM	Good (number is not included)	0.99	No information
^31^	Chemiresistive sensor	(1–1000) ppm	46.98%	Good (no information about R^2^)	1.54 ppm
^32^	Cyclic voltammetry	1 µM to 10 mM	8.39–214.48 μA/mM	R^2^ > 0.97	0.1 µM
This work	Enzymatic sensor (Amperometry)	1 µM to 25 mM (0.0366–913.8)ppm	(7.76–8426) nA/mM	0.92 to 0.98	0.03 µM

## Conclusion and future work

In this research, an enzymatic acetone sensor based on a solid-state electrolyte was developed. Cyclic voltammetry and amperometry were used with disposable platinum electrodes featuring various surface modifications. The sensor demonstrated excellent linear responses across a wide range (1 µM to 25 mM) for both liquid and vapor samples, with a broad sensitivity range.

For liquid samples, the sensor showed sensitivity ranging from 355 to 8426 nA/mM and correlation coefficients ranging from 0.947 to 0.982 for concentrations between 1 and 500 µM, and sensitivity ranging from 8.98 to 38.64 nA/mM with correlation coefficients from 0.944 to 0.98 for concentrations between 1 and 25 mM. When analyzing vapor samples, the sensor exhibited sensitivity ranging from 332 to 1056 nA/mM and correlation coefficients ranging from 0.92 to 0.96 for concentrations between 1 and 500 µM, and sensitivity ranging from 7.76 to 38.8 nA/mM with correlation coefficients from 0.94 to 0.99 for concentrations between 1 and 25 mM. The sensor demonstrated a fast response time of 30–50 s and an impressive limit of detection as low as 0.03 µM.

The results showed that the sensor's performance was optimal with the four-layer surface modification (N + N + E + N). This surface modification will be used for the development of a future breath acetone detection device that can be used for diabetes monitoring.

This research highlights Nafion's effectiveness as a solid-state electrolyte for enzymatic acetone sensing, making it suitable for integration into multiplexed sensing applications. This simplifies the development of handheld devices, especially for diabetes monitoring.

Given the enzymatic nature of this sensor, precise calibration is imperative because the enzyme's lactate content depletes during sensor operation. Complete lactate consumption would hinder sensor reusability. Hence, our approach involved commencing with low concentrations and gradually increasing them to ensure continuous enzyme availability, thus facilitating sustained sensor functionality across various concentrations. Despite encountering inaccuracies in certain trends, we addressed such instances by introducing a fresh electrode to ensure comprehensive coverage of the concentration spectrum and validate trends through experimentation. Ultimately, we maintain confidence in the accuracy and practicality of our calibration method, particularly considering the disposable nature of this sensor. In practical scenarios, like measuring breath acetone, the sensor will be employed for a single sample and situation, similar to the usage of blood glucose strips.

In the future, a complete device incorporating an enzyme-based acetone sensor will be designed for measuring breath acetone within the physiological range, offering potential benefits for diabetes monitoring. As part of our ongoing work, some interference tests have been already performed, which include detecting CO_2_, O_2_, water vapor, methane, ethanol, and hydrogen. Initial results indicate that the acetone sensor remains stable even after exposure to these interference gases, showing no noticeable changes in response to varying concentrations of these interfering substances.

## Data Availability

All data generated or analyzed during this study are included in this published article.
